# Relationship between vascular ageing and left ventricular geometry in patients with newly diagnosed primary aldosteronism

**DOI:** 10.3389/fendo.2022.961882

**Published:** 2022-08-08

**Authors:** Miao Huang, Jiaying Li, Xiexiong Zhao, Shunsong Chen, Xiaogang Li, Weihong Jiang

**Affiliations:** Department of Cardiology, The Third Xiangya Hospital, Central South University, Changsha, China

**Keywords:** vascular ageing, ankle–brachial index, brachial–ankle pulse wave velocity, carotid intima-media thickness, left ventricular geometry, primary aldosteronism

## Abstract

**Background:**

Changes in left ventricular (LV) geometry are early manifestations of cardiac damage. The relationship between vascular aging and LV geometry has been reported. However, in newly diagnosed primary aldosteronism (PA), with more severe target organ damage than essential hypertension, the relationship between vascular aging and LV geometry has never been described.

**Methods:**

We conducted a retrospective study among newly diagnosed PA from 1 January 2017 to 30 September 2021 at the Third Xiangya Hospital. The data of vascular aging parameters were collected, including ankle–brachial index (ABI), brachial–ankle pulse wave velocity (baPWV), and carotid intima-media thickness (cIMT). Echocardiography data were collected to assess LV geometry patterns.

**Results:**

A total of 146 patients with newly diagnosed PA were included. The mean age was 44.77 ± 9.79 years, and 46.58% participants were women. Linear regression analysis adjusting all potential confounders showed that cIMT was significantly associated with LV mass index (LVMI) (β=0.164, P=0.028) and baPWV was significantly associated with relative wall thickness (RWT) (β= 0.00005, P=0.025). Multifactorial adjusted logistic regression analysis demonstrated that cIMT was significantly associated with LV hypertrophy (LVH) (OR=7.421, 95%CI: 1.717–815.688, P=0.021) and baPWV was significantly associated with LV concentric geometry (LVCG) (OR=1.003, 95%CI: 1.001–1.006, P=0.017).

**Conclusion:**

baPWV was significantly associated with LVCG and cIMT was significantly associated with LVH in newly diagnosed PA. This study provides insights on the importance of baPWV measurement and cIMT measurement in early assessment of cardiac damage in newly diagnosed PA.

## Introduction

Primary aldosteronism (PA) is defined as increased secretion of aldosterone by the adrenal cortex and suppressed activity of the renin–angiotensin system, which is clinically manifested as hypertension and hypokalemia (1). The prevalence of PA varied according to the population studied: it ranged from 3.2% to 12.7% in primary care and from 1% to 29.8% in referral centers (2, 3). Studies have demonstrated that patients with PA have more worse target organs of the heart and kidneys compared to patients with essential hypertension (4–7). Changes in left ventricular (LV) geometry, as an important target organ injury induced by elevated blood pressure, are independent predictors of cardiovascular events (8, 9). Early identification of the potential risk factors of changes in LV geometry plays an important role in preventing cardiovascular disease.

Vascular aging reflects the structural and functional changes of the large conduit arteries (10). Carotid intima-media thickness (cIMT), brachial–ankle pulse wave velocity (baPWV), and ankle–brachial index (ABI) are main evaluation indicators to detect the degree of vascular aging (11). The relationship between vascular aging and LV geometry patterns has been reported in a previous study. A recent study based on Northern Shanghai general populations revealed that baPWV, carotid-femoral PWV (cfPWV), and cIMT were associated with structural measurements of LV including relative wall thickness (RWT) with LV mass index (LVMI), and increased cfPWV and increased baPWV and decreased ABI were significantly associated with LV concentric geometry (LVCG) (12). A previous study found that cfPWV and baPWV were significantly correlated with LV hypertrophy (LVH) in a community-based elderly cohort (13). A prospective cohort study showed that baPWV had significant correlations with RWT and LVMI in untreated hypertensive patients, and baPWV was significantly increased in patients with LVH (14). Now, available studies are based on data from the general population and essential hypertension.

However, in PA, a population at high risk for cardiovascular events, the relationship between vascular aging and LV geometry has never been explored. In the present study, we aimed to investigate the association of the vascular aging parameter (baPWV, ABI, and cIMT) and LV geometry in new diagnosed patients with PA and to provide support for the assessment of target organ injury in the preliminary evaluation of PA.

## Methods

### Study population

The study participants were recruited from 1 January 2017 to 30 September 2021 at the Department of Cardiology, The Third Xiangya Hospital, Central South University, Changsha, China. The records of 146 patients with newly diagnosed PA were retrospectively collected in the database of the Third Xiangya Hospital. The inclusion criteria were as follows: (1) age ≥18 years old and (2) confirmed diagnosed with PA on this admission and not previously diagnosed with PA. The exclusion criteria were as follows: (1) missing echocardiography data; (2) simultaneously missing data of baPWV, ABI, and cIMT; (3) a definite diagnosis of other secondary hypertension; (4) serious heart disease (New York Heart Association functional classification ≥III) or severe chronic kidney disease [estimated glomerular filtration rate (eGFR) < 30 ml/min/1.73 m^2^]; (5) cardiovascular events within the last 6 months, including myocardial infarction or stroke; (6) suffered from cancer and other systemic diseases, including acute or chronic inflammatory diseases; and (7) pregnant or lactating women. The present study was approved by the Medical Ethics Committee of the Third Xiangya Hospital (Approval ID: I22013). All the patients signed informed consent at admission and agreed to share their health information for medical research.

### Screening and confirmatory tests for primary aldosteronism

Before screening and confirmatory tests, antihypertensive medicine with great influence on the aldosterone-to-renin ratio (ARR) was withdrawn at least 4 weeks according to the Endocrine Society’s clinical practice guideline (15). Patients with poor blood pressure control may be treated with alpha blockers or non-dihydropyridine calcium channel blockers, which have a lesser effect on ARR. Blood samples were collected to measure plasma aldosterone concentration (PAC) and plasma renin activity (PRA) in the morning after patients woke up and maintained a non-supine position (sitting, standing, or walking) for at least 2 h and sitting for 5–15 min. ARR was calculated as PAC/PRA. For patients with positive screening (ARR>20 ng·ml^-1^/ng·ml^-1^·h^-1^), further confirmatory tests were performed to confirm the diagnosis.

All patients with positive screening tests underwent seated saline suppression testing (SSST). PAC was measured basally at 08:00 and after the completion of an infusion of 2 L of normal saline in 4 h, and PA was diagnosed when PAC >10 ng·ml^-1^ after the infusion of 2 L of normal saline (15). Studies have confirmed that SSST is more sensitive than recumbent saline suppression testing (RSST) in the diagnosis of PA (16).

### Data collection and variable definitions

Information on sociodemographic characteristics and clinical data were obtained from electronic medical records, including age, gender, height, weight, smoking status (current, never, or past), drinking status (current, never, or past), serum potassium level, blood pressure (BP), heart rate, the duration of hypertension, a family history of premature cardiovascular disease, and BP-lowing drug treatment. BP measurements were taken in the sitting position after a rest period of at least 5 min using an automated electronic device (Omron HEM-7200, OmronCo, Dalian, China). The body mass index (BMI) is calculated by dividing the body weight in kilograms by the square of the height in meters. Biochemical parameters included fasting blood glucose (FBG), blood lipid levels (total cholesterol, triglycerides, high-density lipid cholesterol, and low-density lipid cholesterol), and serum creatinine. The eGFR was calculated using the Modification of Diet in Renal Disease formula (17).

### Measurement of vascular aging parameters

The ABI and baPWV were measured simultaneously with an automatic waveform analyzer (BP-203 RPE III; Omron, Dalian, China). The measurement was taken in the supine position after a rest period of at least 5 min. ABI was measured as the ratio of the systolic BP of the ankle artery to that of the brachial artery. The pulse waves of the brachial and posterior tibial arteries of the left and right limbs were measured to assess the transmission time between brachial and posterior tibial artery waveforms. The baPWV was calculated as the brachial–ankle distance divided by the transmission time (18). The average values of the left- and right-side assessments were calculated and used for analysis. ABI and baPWV were measured in 105 patients. Carotid ultrasonography was performed to measure cIMT using color Doppler ultrasound (Vivid E95; GE Healthcare, Massachusetts (MA), USA; or EPIQ 7C; Philips Medical Systems, Massachusetts (MA), USA). The cIMT represents the distance between two parallel echogenic lines corresponding to the lumen-intima interface and media-adventitia interface (19). The cIMT value used for analysis was defined as the average value of the left and right assessment. The cTMT was measured in 135 patients.

### Echocardiography assessment of left ventricular geometry

Echocardiography was performed by trained cardiologists using an available machine (Vivid E95; GE Healthcare, MA, USA or EPIQ 7C; Philips Medical Systems, MA, USA), according to the recommendation from the American Society of Echocardiography and the European Association of Cardiovascular Imaging (20). The LV internal diameter (LVID) at the end-diastole, interventricular septum (IVS), and posterior wall thickness (PWT) at the end-diastole were measured at the standard parasternal window. LV mass was calculated by a validated formula (20). LVMI was calculated with the formula (LV mass/body surface area) and RWT with the formula (2 × PWT/LVID) (20, 21).

LV geometry patterns were defined into normal, concentric remodeling, concentric LVH, and eccentric LVH. LVH was defined as LVMI >115 g/m² in men and >95 g/m² in women, including concentric LVH (RWT > 0.42), and eccentric LVH (RWT ≤ 0.42). RWT > 0.42 without LVH was considered diagnostic for concentric remodeling. Concentric LVH and concentric remodeling were defined as LVCG.

### Statistical analysis

Continuous variables were presented as weighted means ± standard deviation or median (interquartile range) and categorical variables as numbers (percentages). Continuous variables were compared using Student’s t-test or Wilcoxon rank sum test, and categorical variables were compared using chi-square analysis. Spearman correlation analysis was performed to assess the correlation of vascular ageing parameters with LVMI and RWT. The linear regression model was applied to compare the relationship between vascular aging parameters with LVMI and RTW. When the dependent variable was not normally distributed data, we performed logarithmic transformation on the data. When the association of vascular ageing parameters and LV geometry was investigated, logistic regression analysis was used to calculate odds ratios (ORs) and 95% confidence intervals (95%CIs) by controlling age, gender, BMI, smoking status, drinking status, BP, heart rate, FBG, TG, HDL-C, eGFR, serum potassium level, PAC, the duration of hypertension, a family history of premature cardiovascular disease, and BP-lowering drug treatment. All statistical analyses were performed using Stata version 16.0 (StataCorp, College Station, TX, USA). P-value < 0.05 (two sided) was considered to be statistically significant.

## Results

### Characteristics of study patients

This study included 146 patients with newly diagnosed PA. The characteristics of these patients are shown in **Table 1**. The participants’ mean age was 44.77 ± 9.79 years, and 46.58% of participants were female, with an elevated median value of systolic BP (160 mm Hg) and diastolic BP (96 mmHg). The duration of the hypertension of patients was 2.00 years, and approximately 60% of the patients were receiving BP-lowering drug treatment. The median of the baPWV value and cIMT value were 1,618 cm/s and 0.9 mm, respectively. The mean value of ABI was 1.15. Approximately 87.67% of the patients had abnormal LV geometry patterns. Approximately 50.68% of patients had LVH, and 83.56% had LVCG.

**Table 1:** Characteristics of study patients.

Variables	Value
**Age (years)**	44.77 ± 9.79
**Female, n(%)**	68(46.58)
**BMI (kg/m2)**	25.41 ± 3.60
**Current smoking, n(%)**	33(22.60)
**Current drinking, n(%)**	13(8.90)
**Systolic blood pressure (mmHg)**	160(143–170)
**Diastolic blood pressure (mmHg)**	96(87–106)
**Heart rate (beats/min)**	71(65–76)
**TC (mmol/L)**	4.31 ± 0.92
**TG (mmol/L)**	1.55(0.99–2.35)
**LDL-C (mmol/L)**	2.25 ± 0.67
**HDL-C (mmol/L)**	1.10(0.95–1.27)
**FBG (mmol/L)**	5.02(4.54–5.44)
**eGFR (ml/min/1.73 m^2^)**	99.14(85.07–112.98)
**Serum K^+^ (mmol/L)**	3.51 ± 0.52
**PAC (ng/ml)**	29.62(25.08–33.55)
**PRA (ng ml^-1^/h)**	0.89(0.74–1.17)
**ARR (ng ml^-1^/ng ml^-1^ h^-1^)**	31.82(25.43–37.50)
**Family history of premature CVD, n(%)**	48(32.88)
**Duration of hypertension, year**	2.00(0.25–5.00)
**Blood pressure–lowering drug treatment, n(%)**	89(60.96)
**baPWV (cm/s)**	1,618(1,456–1,830)
**ABI**	1.15 ± 0.07
**cIMT (mm)**	0.90(0.75–1.05)
**LVMI (g/m^2^)**	104.67(92.39–124.24)
**RWT**	0.49 ± 0.07
**Left ventricular geometry patterns**	
Normal	18(12.33)
Concentric remodeling	54(36.99)
Concentric LVH	68(46.57)
Eccentric LVH	6(4.11)
**LVH**	74(50.68)
**LVCG**	122(83.56)

Continuous variables were presented as weighted means ± standard deviation or median (interquartile range) and categorical variables as numbers (percentages).

BMI, body mass index; TC, total cholesterol; TG, triglycerides; LDL-C, low-density lipid cholesterol; HDL-C, high-density lipid cholesterol; FBG, fasting blood glucose; eGFR, estimated glomerular filtration rate; CVD, cardiovascular disease; PAC, plasma aldosterone concentration; PRA, plasma renin activity; ARR, aldosterone-to-renin ratio; baPWV, brachial–ankle pulse wave velocity; ABI, ankle–brachial index; cIMT, carotid intima-media thickness; LVMI, left ventricular mass index; RWT, relative wall thickness; LVH, left ventricular hypertrophy; LVCG, left ventricular concentric geometry.

### Correlation of vascular parameters with left ventricular mass index and relative wall thickness

**Figure 1:**
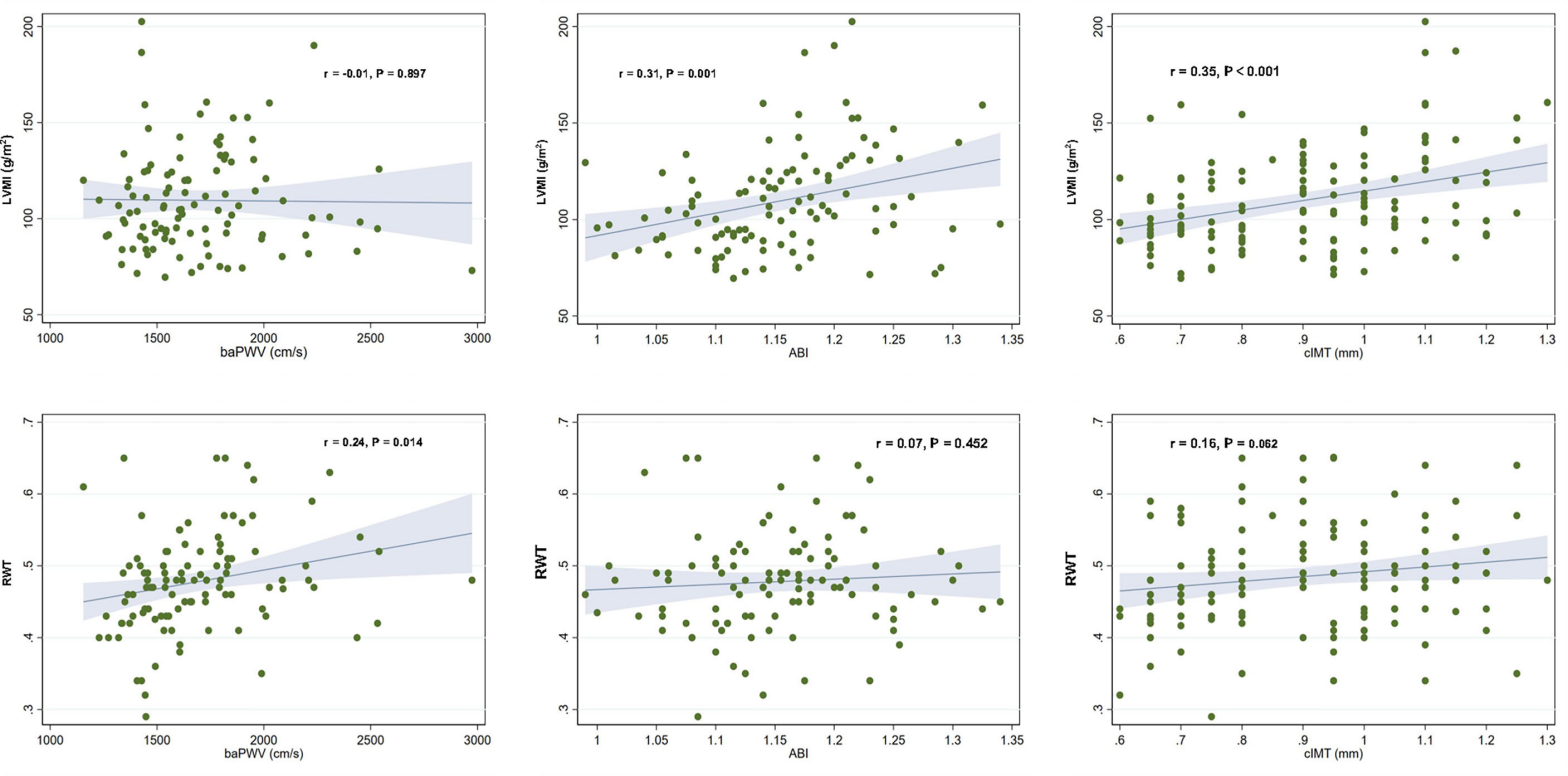
Correlation plots showing the associations of vascular parameters with left ventricular mass index and relative wall thickness. Each dot indicates an individual patient’s data. The linear regression line (blue line) and 95% confidence interval (shaded area) are depicted. LVMI, left ventricular mass index; baPWV, brachial–ankle pulse wave velocity; ABI, ankle–brachial index; cIMT, carotid artery intima-media thickness; RWT, relative wall thickness.

The correlation of vascular parameters with LVMI and RWT is demonstrated in **Table 2**. ABI (r=0.313, P=0.001) and cIMT (r=0.346, P<0.001) were positively correlated with LVMI. BaPWV was positively correlated with RWT (r=0.238, P=0.014). The correlation plots of vascular parameters with LVMI and RWT are shown in **Figure 1**.

**Table 2:** Correlation of vascular parameters with LVMI and RWT.

Parameters	LVMI		RWT	
	r	P	r	P
baPWV(cm/s)	-0.013	0.897	0.238	0.014
ABI	0.313	0.001	0.074	0.452
cIMT(mm)	0.346	<0.001	0.161	0.062

baPWV, brachial–ankle pulse wave velocity; ABI, ankle–brachial index; cIMT, carotid intima-media thickness.

### Association of vascular parameters with left ventricular mass index and relative wall thickness

The results of the linear regression analysis of the relationship of vascular parameters with LVMI and RWT are summarized in **Table 3**.** A** crude model showed that ABI and cIMT were positively associated with LVMI and baPWV was positively associated with RWT. After adjusting all potential confounders, cIMT was still significantly associated with LVMI (β=0.164, P=0.028) and baPWV was still significantly associated with RWT (β= 0.00005, P=0.025).

**Table 3:** Linear regression analysis showing the association of LVMI and RWT with vascular parameters.

	Crude Model^a^		Adjusted Model^b^	
	β(95%CI)	P	β(95%CI)	P
**logLVMI**				
baPWV(cm/s)	0.00004(-0.00005–0.00013)	0.377	0.00003(-0.00005–0.00012)	0.436
ABI	0.392(0.010–0.773)	0.044	0.041(-0.340–0.423)	0.830
cIMT(mm)	0.196(0.066–0.326)	0.003	0.164(0.018–0.311)	0.028
**RWT**				
baPWV(cm/s)	0.00005(0.00001–0.00009)	0.014	0.00005(0.00001–0.00011)	0.025
ABI	0.073(-0.118–0.263)	0.452	-0.053(-0.268–0.161)	0.623
cIMT(mm)	0.067(-0.003–0.137)	0.062	0.044(-0.043–0.131)	0.316

a without adjustment.

b adjusted for age, gender, body mass index, smoking status, drinking status, blood pressure, heart rate, triglycerides, high-density lipid cholesterol, fasting blood glucose, estimated glomerular filtration rate, serum potassium level, plasma aldosterone concentration, the duration of hypertension, a family history of premature cardiovascular disease, and blood pressure–lowering drug treatment.

LVMI, left ventricular mass index; baPWV, brachial–ankle pulse wave velocity; ABI, ankle–brachial index; cIMT, carotid intima-media thickness; RWT, relative wall thickness.

### Association of vascular parameters with left ventricular hypertrophy and left ventricular concentric geometry

**Figure 2:**
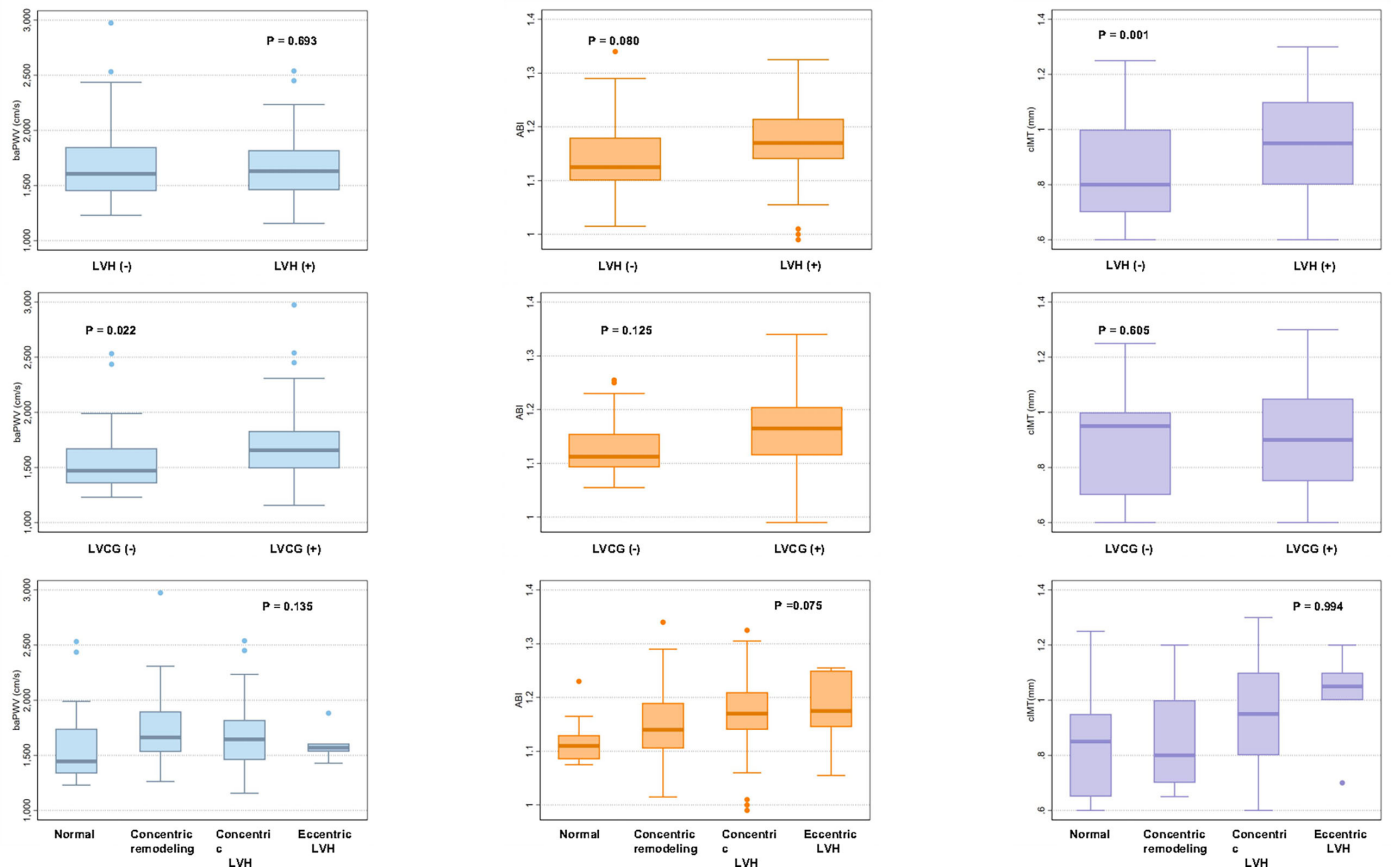
Box plots showing brachial–ankle pulse wave velocity, ankle–brachial index, and carotid intima-media thickness by left ventricular geometry patterns. BaPWV, ABI and cIMT values are shown as box plots with the median and interquartile range. BaPWV, brachial–ankle pulse wave velocity; ABI, ankle–brachial index; cIMT, carotid intima-media thickness; LVH, left ventricular hypertrophy; LVCG, left ventricular concentric geometry.

We compared the values of vascular parameters between LVH and non-LVH and LVCG and non-LVCG. As shown in **Figure 2**, patients with LVH had significantly higher cIMT values compared to those without LVH (P=0.001), and patients with LVCG had significantly higher baPWV values compared to those without LVCG (P=0.022).We further stratified the values of vascular parameters into four groups of LV geometry patterns. There was no statistical difference in the values of vascular parameters between the four LV geometry patterns.

**Table 4** shows the logistic regression analysis results of the association of vascular parameters with LVH and LVCG. The crude model showed that cIMT was positively associated with LVH and baPWV was positively associated with LVCG. After adjusting all potential confounders, cIMT was still significantly associated with LVH (OR=37.421, 95%CI: 1.717–815.688, P=0.021) and baPWV was still significantly associated with LVCG (OR=1.003, 95%CI: 1.001–1.006, P=0.017).

**Table 4:** Logistic regression analysis of LVH and LVCG with vascular parameters.

	Crude Model^a^		Adjusted Model^b^	
	β(95%CI)	P	β(95%CI)	P
**LVH**				
baPWV(cm/s)	1.000(0.999–1.001)	0.775	0.998(0.996–1.000)	0.074
ABI	142.149(0.520–38870.310)	0.083	1.601(0.001–4107.266)	0.906
cIMT(mm)	26.480(3.375–207.745)	0.002	37.421(1.717–815.688)	0.022
**LVCG**				
baPWV(cm/s)	1.001(1.000–1.003)	0.045	1.003(1.001–1.006)	0.017
ABI	278.184(0.202–382199.400)	0.127	82.108(0.004–1824029.000)	0.388
cIMT(mm)	1.879(0.153–23.021)	0.622	2.853(0.092–88.122)	0.549

a without adjustment.

b adjusted for age, gender, body mass index, smoking status, drinking status, blood pressure, heart rate, triglycerides, high-density lipid cholesterol, fasting blood glucose, estimated glomerular filtration rate, serum potassium level, plasma aldosterone concentration, the duration of hypertension, a family history of premature cardiovascular disease, and blood pressure–lowering drug treatment.

LVH, left ventricular hypertrophy; baPWV, brachial–ankle pulse wave velocity; ABI, ankle–brachial index; cIMT, carotid intima-media thickness; LVCG, left ventricular concentric geometry.

## Discussion

To the best of our knowledge, this is the first study to explore the relationship of baPWV, ABI, and cIMT with LV geometry in patients with newly diagnosed PA. In the present study, we found that baPWV was significantly associated with RWT and LVCG and cIMT was significantly associated with LVMI and LVH in newly diagnosed PA. The finding of this study provided evidence for the significant contribution of baPWV and cIMT to abnormal LV geometry in newly diagnosed PA.

The change of LV geometry is a response to systemic and local hemodynamic changes and has also been described as target organ injury (22, 23). It is widely recognized that LV geometry changes are independent risk factors for cardiovascular events (24–26). A number of studies have examined the relationship between vascular aging parameters and LV geometry and found that they are closely related (12–14, 27–34). The Northern Shanghai Study (NSS) involving a community-dwelling older population suggested that baPWV, cfPWV, and cIMT were significantly associated with LVMI and RWT, and cIMT was significantly related to LVMI, even adjusting for conventional cardiovascular risk factors and diseases and treatments (12). A study investigated the association of baPWV with LV geometry in treatment-naive hypertensive patients, which found that baPWV had significant associations with RWT and LVMI and showed a fair discrimination ability of LVH (14). There were two studies that have assessed the relationship between cIMT and LV geometry in hypertensive patients (27, 28). A study on Italian hypertensive patients showed a significant correlation of cIMT and LVMI (28), and another study on Korean hypertensive patients found that cIMT was independently associated with RWT (20). Linde et al. demonstrated that increased cfPWV was also associated with LVH in non-elderly ischemic stroke survivors (32). However, those epidemiological results on the relationship of the vascular aging parameter and LV geometry were based on the general population or hypertensive patients.

In this current study, we analyzed the association of baPWV, ABI, and cIMT with LV geometry in patients with newly diagnosed PA. Our study found that baPWV was positively correlated with RWT; ABI and cIMT were positively correlated with LVMI, which was consistent with previous studies (12, 28). Moreover, we found that when the potential covariables were adjusted, baPWV was still significantly associated with RWT and cIMT was still significantly associated with LVMI. Based on these findings, we analyzed the association of vascular aging parameters with LVH and LVCG; we found the significant association of baPWV with LVCG and cIMT with LVH. In untreated hypertensive patients, baPWV showed a significant association of LVH (14); however, our research did not find that baPWV was associated with LVH. The Possible causes were considered: there were confounding factors between the association of baPWV and LVH and the population in this study was from a single center.

Vascular remodeling in patients with PA mainly involves the increase of cells and extracellular matrix in the subintimal space and middle layer, which is manifested by vascular wall thickening, arterial stiffness, and subsequent vascular dysfunction (35, 36). BaPWV is an evaluation of arterial stiffness and reflects the elasticity of middle-sized and large arteries (37–39); cIMT is more reflective of the arterial wall structure throughout the body (40). A novel finding of our study is that baPWV was significantly associated with LVCG and cIMT was significantly associated with LVH in newly diagnosed PA. The study will provide a preliminary evidence of the association of the vascular aging parameter and LV geometry in patients with newly diagnosed PA. It is suggested that in newly diagnosed PA, early baPWV measurement has important clinical significance in assessing LV geometry changes and carotid vascular ultrasound in assessing LVH. The most plausible explanation for the association between vascular aging parameters and LV remodeling is that arterial stiffness and systemic changes in the vascular wall structure reflect the pressure pulse wave back to the LV more quickly, resulting in increased afterload of LV (41–43).

This study also has some limitations. First, since this study was retrospective, it could not provide a causal relationship between vascular parameters and LV geometry. Future studies could follow up the effects of increased baPWV or cIMT on LV geometry using a longitudinal design. Furthermore, we used baPWV as the indicator of arterial stiffness, while cfPWV is considered a gold indicator of arterial stiffness (18). However, the value of baPWV has been shown to be strongly associated with the value of cfPWV (44) and baPWV was better correlated with LV mass than cfPWV (45), suggesting baPWV as an acceptable indicator of vascular stiffness. In addition, our study participants were from a single center and the inclusion was Chinese patients, so that our results cannot be generalized to other groups of subjects with different demographics.

## Conclusion

The present study suggested that baPWV was significantly and independently associated with LVCG and cIMT was significantly and independently associated with LVH in patients with newly diagnosed PA. This study provides insights on the importance of early baPWV and cIMT measurement in the assessment of LV geometry change in newly diagnosed PA.

## Data availability statement

The raw data supporting the conclusions of this article will be made available by the authors, without undue reservation.

## Ethics statement

This study was reviewed and approved by the Medical Ethics Committee of the Third Xiangya Hospital. The patients/participants provided their written informed consent to participate in this study.

## Author contributions

WJ and XL contributed to conception and design of the study. MH and JL wrote the first draft of the manuscript. MH and JL performed the statistical analysis. XZ and SC wrote the sections of the manuscript. All authors contributed to article and approved the submitted version.

## Funding

The study was supported by the Key Research and Development program of Hunan Province (NO.2022SK2029), the National Natural Science Foundation of China Projects (NO.81800271).

## Conflict of interest

The authors declare that the research was conducted in the absence of any commercial or financial relationships that could be construed as a potential conflict of interest.

## Publisher’s note

All claims expressed in this article are solely those of the authors and do not necessarily represent those of their affiliated organizations, or those of the publisher, the editors and the reviewers. Any product that may be evaluated in this article, or claim that may be made by its manufacturer, is not guaranteed or endorsed by the publisher.
